# Blood Homocysteine Levels Mediate the Association Between Blood Lead Levels and Cardiovascular Mortality

**DOI:** 10.1007/s12012-023-09819-0

**Published:** 2024-01-17

**Authors:** Sapha Shibeeb, Atiyeh Abdallah, Zumin Shi

**Affiliations:** 1 School of Health and Biomedical Sciences, RMIT University, PO Box 71, Bundoora, Melbourne, VIC 3083 Australia; 2 Department of Biomedical Sciences, College of Health Sciences, QU Health, Qatar University, Doha, Qatar; 3 Human Nutrition Department, College of Health Sciences, QU Health, Qatar University, Doha, Qatar

**Keywords:** Lead exposure, Homocysteine, Cardiovascular diseases, Mortality, NHANES

## Abstract

Lead is a heavy, toxic metal and its exposure to humans can lead to increased risk of cardiovascular disease development and mortality. Lead exposure has been shown to induce hyperhomocysteinemia (HHCy) which may be a major pathogenic risk for the risk of CVDs. The aim of this study was to investigate whether homocysteine (Hcy) mediates the effect of lead on cardiovascular mortality. A total of 17,915 adults aged ≥ 20 who participated in the National Health and Nutrition Examination Survey (1999 to 2006). Information on mortality was ascertained via probabilistic matching to the death certificates from the National Death Index recorded up to December 31, 2015. Cox proportional hazards regression was performed to assess the association between blood lead levels and mortality. Mediation via Hcy was examined using a logit model. During a mean follow-up of 11.6 years, the incidences of CVD mortality were 0.73, 2.18, 3.03 and 4.94 per 1000 person-years across quarterlies of blood lead levels from low to high. Following multivariable adjustment, blood lead levels were strongly associated with CVD mortality in all mortality models (p-trend < 0.001). This association remained statistically significant after further adjusting for quartiles of homocysteine (model 3; HR 1.38 (95% CI 1.01—1.89) p-trend < 0.001). Furthermore, blood lead levels increased the odds of CVD mortality via homocysteine (indirect effect) (OR 1.42 (95% CI 1.30—1.55)), demonstrating the mediatory effect of homocysteine. This the first study that demonstrates that increased homocysteine mediates nearly half of CVD mortality related to blood lead levels.

## Introduction

Cardiovascular disease (CVD) remains the number one cause of death worldwide, accounting for over two-thirds of all mortality [1, 2]. Over the last few decades, CVD mortality rates have decreased across the age spectrum in the US population [3], however, in the US, heart diseases remain the leading cause of death for both men and women [4]. CVD describes a large group of genetic and acquired diseases affecting the heart and blood vessels, including congenital heart and blood vessel disorders, atherosclerosis, valve disease, and arrhythmias [5]. As a multifactorial disease, there are many genetic and environmental risk factors for CVD: genetic factors include mutations, polymorphisms, and epigenetic alterations [6–8], while common environmental risk factors include diet, smoking, alcohol consumption, and pollution [2, 9]. Of the latter, lead exposure confers a high risk for developing CVD, and high blood lead levels (BLL) are associated with high CVD mortality rates [10–12].

Although preventative public health measures mean that the risk of acquiring high BLL in the US has decreased [13], large-scale sources of lead contamination still exist in the air, soil, and water [14]. Lead is a heavy metal that is toxic to humans. There is a consensus that a BLL of 10 µg/dL in adults and 3.5 µg/dL in children represent “high” BLL. However, the Advisory Committee on Childhood Lead Poisoning Prevention (ACCLPP) of the Centers for Disease Control and Prevention (CDC) indicate that any circulating lead may be toxic [15]. Recently, CDC recommended revising the upper limit of the reference value to 3.5 μg/dL, furthermore, a recent study reported that more than 170 million individuals in the US were exposed to over 5 μg/dL during childhood [16]. Lead can inhibit many enzymes and induce oxidative stress by forming complexes with proteins, amino acids, and thiol-containing compounds [17]. It increases reactive oxygen species (ROS), which damage biomolecules including DNA and proteins [18] to induce pathophysiological changes that affect the nervous, reproductive, hematopoietic, and cardiovascular systems.

Homocysteine is an amino acid produced during proteolysis, blood homocysteine levels vary between men and women, with a normal range typically between 5 to 15 µmol/L. HHcy is when blood homocysteine levels exceed 15 µmol/L [19]. Lead exposure is associated with elevated homocysteine levels through its interaction with different proteins and disruption of homocysteine metabolism [20, 21]. The association between BLL and HHcy was confirmed in an analysis of over 9,331 participants in the US National Health and Nutrition Examination Survey (NHANES) database [22]. Interestingly, products of homocysteine metabolism such as methionine reduce the damage caused by ROS in cells exposed to lead [23]. Over 100 diseases have now been linked to HHcy, the most common diseases being CVD and neurological diseases [24]. HHcy can contribute to the development of CVDs via through various mechanisms, including damage to the vascular endothelium and smooth muscle cells, leading to changes in arterial structure and function [19].

To our knowledge, there has yet to be a study of whether homocysteine levels mediate the effects of BLL on cardiovascular mortality. Therefore, we examined these associations using National Health and Nutrition Examination Survey study data collected between 1999 and 2006. The study will analyse large sample size and relatively long follow-up duration as well as the representative US sample.

## Methods

### Study Population

**Fig. 1:**
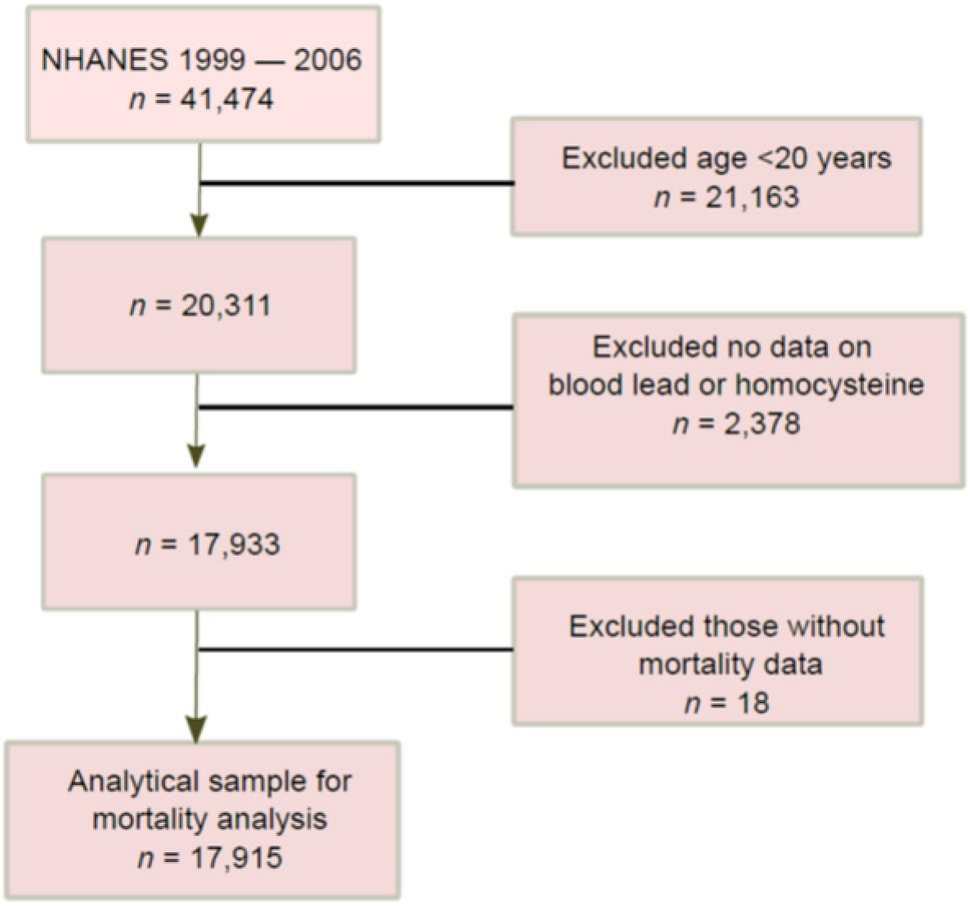
Sample flowchart

This study used publicly available data from years (1999–2006) of National Health and Nutrition Examination Survey (NHANES). A total of 17,915 adults aged ≥ 20 years were included in the current analysis after excluding those without blood lead and homocysteine measurements (Fig. 1). The NHANES is carried out annually using a complex multistage study design to assess the health and nutritional status of the non-institutionalized US general population. Detailed NHANES protocols and procedures are available elsewhere [10].. In brief, a face-to-face interview was conducted, during which participants were asked to complete questionnaires, undergo medical examination and provide venous blood and other biological samples.

### Data Collection

Blood lead was measured at the Environmental Health Sciences Laboratory of the CDC National Center for Environmental Health (NCEH). Lead levels in whole blood were measured on an inductively coupled plasma mass spectrometry (PerkinElmer, Norwalk, CT) [22]. Homocysteine was measured by a fully automated fluorescence polarization immunoassay (Abbott Diagnostics, Abbott Park, IL). Other data collected include sociodemographic characteristics, including age, sex, race/ethnicity, marital status, education, physical activity, current smoking, alcohol intake and poverty income ratio.

### Population Attributable Fraction

In the present study, we calculated the population attributable fraction (PAF) using the formula: PAF = Pe * (RR–1)/(Pe * (RR–1) + 1), wherein ‘Pe’ denotes the prevalence of elevated blood lead (or highest quartile of blood lead). We defined elevated blood lead as blood lead ≥ 3.5 µg/L.

### Mortality

Information on mortality was ascertained via probabilistic matching to the death certificates from the National Death Index recorded up to December 31, 2015. The cause of death was coded according to the International Classification of Diseases, Tenth Revision (ICD-10). In the current analysis, CVD mortality was defined based on ICD codes I00–I99, while cancer-mortality was based on ICD codes C00–C97. This method has been validated and widely used [25, 26].

### Statistical Analysis

Blood lead level was categorised into quartiles with quartile 1 (Q1) and quartile 4 (Q4) as the lowest and highest, respectively. Sample characteristics was presented as mean (SD) or as percentages and the differences in subject characteristics were tested using one-way analysis of variance (ANOVA) or chi-square tests. Survey weight was used in all the multivariable analyses, except the mediation analysis. Follow-up duration was calculated as the difference between the survey date and the last known date alive or censored from the linked mortality file. Multivariate Cox proportional hazards regression was performed, to assess the association between blood lead levels and mortality. Time on survey was used as the time scale in the Cox regression. Three models were used in Cox regression analyses: model 1 was adjusted for age, gender and race; model 2 was further adjusted for income-poverty-ratio, leisure time physical activity, education, smoking, alcohol drinking and BMI; model 3 was further adjusted for homocysteine. Kaplan–Meier survival analysis was used to determine survival by quartiles of blood lead level. A logit model in a generalization method was used to determine multiple-adjusted odds ratios with 95% confidence intervals (OR [95% CI]) for CVD mortality according to total, direct and indirect (mediation via homocysteine) effects of blood lead levels [27]. All the analyses were performed using STATA (Version 17.0, Stata Corporation, College Station, TX, USA).

## Results

### General Characteristics of Study Participants

A total of 17,915 participants who attended NHANES 1999–2006 were included in this study, with a mean age of 49.5 years. 47.6% of participants were men, and 52.4% were women. Non-Hispanic White participants comprised the majority of the sample size with 50.5% of study participants. The unweighted distributions of population characteristics and other covariates are shown in Table 1.

**Table 1:** Sample characteristics by quartiles of blood lead among participants attending NHANES 1999–2006 (*N* = 17,915)

	Total	Q1	Q2	Q3	Q4	*p*-value
	*N* = 17,915	*N* = 5,078	*N* = 4,297	*N* = 4,109	*N* = 4,431	
Age (years)	49.5 (19.0)	38.4 (16.2)	48.7 (18.1)	54.4 (17.7)	58.4 (17.7)	< 0.001
Gender						< 0.001
Men	8,526 (47.6%)	1,273 (25.1%)	1,903 (44.3%)	2,326 (56.6%)	3,024 (68.2%)	
Women	9,389 (52.4%)	3,805 (74.9%)	2,394 (55.7%)	1,783 (43.4%)	1,407 (31.8%)	
Race						< 0.001
NH White	9,039 (50.5%)	2,661 (52.4%)	2,201 (51.2%)	2,119 (51.6%)	2,058 (46.4%)	
NH Black	3,510 (19.6%)	909 (17.9%)	801 (18.6%)	775 (18.9%)	1,025 (23.1%)	
Mex American	3,962 (22.1%)	1,066 (21.0%)	933 (21.7%)	898 (21.9%)	1,065 (24.0%)	
Other race/ethn	1,404 ( 7.8%)	442 ( 8.7%)	362 ( 8.4%)	317 ( 7.7%)	283 ( 6.4%)	
Education						< 0.001
< 11 grade	5,600 (31.3%)	1,067 (21.0%)	1,241 (28.9%)	1,337 (32.6%)	1,955 (44.3%)	
HS displ or GED	4,244 (23.7%)	1,156 (22.8%)	999 (23.3%)	1,059 (25.8%)	1,030 (23.3%)	
Some college	4,687 (26.2%)	1,660 (32.7%)	1,163 (27.1%)	972 (23.7%)	892 (20.2%)	
> college	3,350 (18.7%)	1,194 (23.5%)	885 (20.6%)	735 (17.9%)	536 (12.1%)	
Smoking						< 0.001
Never	9,252 (51.7%)	3,463 (68.2%)	2,326 (54.2%)	1,858 (45.3%)	1,605 (36.3%)	
Former	4,728 (26.4%)	943 (18.6%)	1,087 (25.3%)	1,232 (30.0%)	1,466 (33.2%)	
Current smoker	3,911 (21.9%)	669 (13.2%)	879 (20.5%)	1,012 (24.7%)	1,351 (30.6%)	
Alcohol drinking						< 0.001
No	3,518 (19.6%)	862 (17.0%)	769 (17.9%)	859 (20.9%)	1,028 (23.2%)	
Yes	10,691 (59.7%)	2,922 (57.5%)	2,579 (60.0%)	2,482 (60.4%)	2,708 (61.1%)	
Missing	3,706 (20.7%)	1,294 (25.5%)	949 (22.1%)	768 (18.7%)	695 (15.7%)	
BMI (kg/m2)	28.4 (6.4)	29.1 (7.2)	28.7 (6.6)	28.4 (5.8)	27.5 (5.4)	< 0.001
Physical activity (METs minutes/week)						< 0.001
< 600 MET/Wk	8,729 (48.7%)	2,234 (44.0%)	2,044 (47.6%)	2,007 (48.8%)	2,444 (55.2%)	
600–1200 MET/Wk	2,616 (14.6%)	843 (16.6%)	638 (14.8%)	596 (14.5%)	539 (12.2%)	
> = 1200 MET/Wk	6,570 (36.7%)	2,001 (39.4%)	1,615 (37.6%)	1,506 (36.7%)	1,448 (32.7%)	
Income to poverty ratio						< 0.001
< 1.30	4,687 (28.3%)	1,194 (24.8%)	1,051 (26.3%)	1,041 (27.6%)	1,401 (35.0%)	
1.3–3.5	6,471 (39.0%)	1,851 (38.4%)	1,480 (37.1%)	1,474 (39.1%)	1,666 (41.6%)	
> 3.5	5,425 (32.7%)	1,775 (36.8%)	1,458 (36.6%)	1,259 (33.4%)	933 (23.3%)	
Lead (ug/dL)	1.7 (× /2.0)	0.7 (× /1.4)	1.4 (× /1.1)	2.1 (× /1.1)	4.1 (× /1.5)	< 0.001
Homocysteine (umol/L)	8.1 (× /1.5)	6.5 (× /1.4)	8.0 (× /1.4)	8.8 (× /1.4)	9.8 (× /1.5)	< 0.001
Log-transformed lead (µg/L)	0.5 (0.7)	-0.3 (0.4)	0.3 (0.1)	0.8 (0.1)	1.4 (0.4)	< 0.001
Log-transformed homocysteine (µmol/L)	2.1 (0.4)	1.9 (0.4)	2.1 (0.3)	2.2 (0.3)	2.3 (0.4)	< 0.001

Data are presented as mean (SD) or geometric mean (× /geometric SD) for continuous measures, and n (%) for categorical measures

There was a positive trend between BLL and homocysteine levels. The mean BLL and homocysteine levels were 0.5 μg/L (SD 0.7) and 2.1 μmol/L (SD 0.4) (log-transformed), respectively. There were significant differences between BLL and homocysteine quartiles: participants in the highest quartile had a BLL of 1.4 μg/dL (SD 0.4) (log-transformed) and a blood homocysteine level of 2.3 µmol/L (SD 0.4) (log-transformed). Furthermore, these participants were older, more likely to be NH White males, have lower educational levels, lower physical activity, and higher alcohol consumption.

### All-Cause Mortality and Survival Rates

During a mean follow-up of 11.6 years (208,506 person-years), 3700 deaths occurred. The incidences of all-cause mortality were 4.40, 9.10, 13.62, and 23.89 per 1000 person-years across BLL quartiles from low to high Table 2.

**Table 2:** Hazard ratios (95%CI) for all-cause and CVD mortality by BLL quartiles among NHANESE participants, 1999–2006 (*N* = 17,915)

	BLL quartiles				
	Q1	Q2	Q3	Q4	*p*-trend
*All-cause mortality*					
Number of cases	387	731	970	1,612	
Incidence rate (per 1000 person-years)	4.40	9.10	13.62	23.89	
Model 1	1.00	1.21 (1.02–1.44)	1.32 (1.13–1.56)	1.98 (1.66–2.37)	< 0.001
Model 2	1.00	1.11 (0.93–1.33)	1.23 (1.04–1.46)	1.64 (1.36–1.98)	< 0.001
Model 2 excluding known CVD	1.00	1.04 (0.83–1.30)	1.25 (1.00–1.56)	1.56 (1.24–1.98)	< 0.001
Model 3	1.00	1.08 (0.90–1.30)	1.17 (0.98–1.38)	1.48 (1.23–1.79)	< 0.001
*CVD mortality*					
Number of cases	77	173	239	383	
Incidence rate (per 1000 person-years)	0.73	2.18	3.03	4.94	
Model 1	1.00	1.51 (1.08–2.10)	1.42 (1.04–1.94)	1.86 (1.38–2.52)	< 0.001
Model 2	1.00	1.41 (0.95–2.08)	1.41 (0.98–2.03)	1.72 (1.20–2.46)	0.002
Model 2 excluding known CVD	1.00	1.34 (0.82–2.21)	1.33 (0.81–2.19)	1.40 (0.93–2.12)	0.175
Model 3	1.00	1.35 (0.92–1.99)	1.31 (0.92–1.87)	1.52 (1.07–2.15)	0.021
Lead (ug/dL)	0.7 (× /1.4)	1.4 (× /1.1)	2.1 (× /1.1)	4.1 (× /1.5)	

Model 1 adjusted for age, gender, and race

Model 2 further adjusted for income-poverty ratio, leisure time physical activity, smoking, alcohol consumption, BMI, hypertension, and diabetes

Model 3 is model 2 further adjusted for homocysteine quartiles

BLL presented as geometric mean (× /geometric SD)

**Fig. 2:**
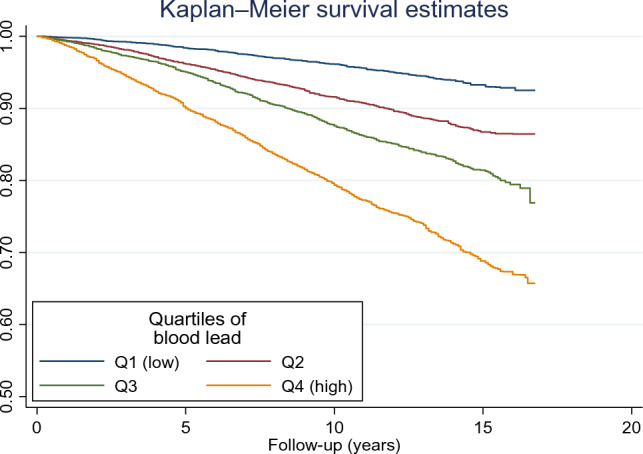
Kaplan–Meier survival curve for all-cause mortality by quartiles of blood lead

There was a dose–response relationship between BLL and all-cause mortality in multivariate-adjusted all-cause mortality models. BLL was associated with higher all-cause mortality in the age, sex, and race-adjusted model (model 1; HR 1.98 (95% CI 1.66–2.37), p-trend < 0.001). The positive relationship remained significant after further adjusting for other demographic characteristics including hypertension and diabetes (model 2; HR 1.64 (95% CI 1.36–1.98), p-trend < 0.001) and excluding known CVD (model 2 excluding known CVD; HR 1.56 (95% CI 1.24–1.98). The relationship remained positive after further adjusting for homocysteine levels (model 3; HR 1.48 (95% CI 1.23–1.79), p-trend < 0.001) (Table 2). Kaplan–Meier survival analysis showed statistically significant lower survival rates for participants with high BLL (1.4 μg/dL, log-transformed; Fig. 2).

### *CVD* Mortality

During a mean follow-up of 11.6 years (208,506 person-years), 872 deaths occurred from CVD. The incidences of CVD mortality were 0.73, 2.18, 3.03, and 4.94 per 1000 person-years across BLL quartiles from low to high (Table 2). Similarly, there was a significant and dose-dependent relationship between BLL and CVD mortality in multivariate-adjusted CVD models (p-trend < 0.001). When adjusted for age, sex, and race, there was a statistically significant association between BLL and CVD mortality (model 1; HR 1.86 (95% CI 1.38–2.52), p-trend < 0.001). This significant association persisted after further adjustments for other demographic characteristics including hypertension and diabetes (model 2; HR 1.72 (95% CI 1.20–2.46), p-trend 0.002) and excluding known CVD (model 2 excluding known CVD; HR 1.40 (95% CI 0.93–2.12). Importantly, this association remained statistically significant after further adjusting for homocysteine (model 4; HR 1.52 (95% CI 1.07–2.15), p-trend < 0.001).

Subgroup analysis for the association between blood lead CVD mortality are reported in Table 3. For women, the highest blood lead quartile showed a statistically significant increased risk of CVD mortality HR (Q4) 1.97 (95% CI: 1.15–3.37), p-value for trend 0.010. A similar pattern was observed among participants with hypertension HR (Q4) 1.77 (95% CI: 1.18–2.66), *p*-value 0.005. Interesting, in participants never smoked there a significantly significant risk of CVD mortality HR (Q4) 1.77 (95% CI: 1.11–2.81), *p*-value for trend 0.010. Furthermore, among current smokers, a statistically significant dose–response relationship observed, with highest quartile having an HR of 3.52 (95% CI: 1.15–10.73), *p*-value for trend 0.025.

**Table 3:** Subgroup analysis for the association between blood lead and CVD mortality

	Quartiles of Pb					
	Q1	Q2	Q3	Q4	p for trend	p for interaction
Gender						0.350
Men	1.00	1.48 (0.76–2.90)	1.29 (0.67–2.49)	1.52 (0.85–2.70)	0.156	
Women	1.00	1.22 (0.74–2.02)	1.47 (0.89–2.40)	1.97 (1.15–3.37)	0.010	
Hypertension						0.603
No	1.00	1.75 (0.69–4.44)	1.68 (0.78–3.63)	1.49 (0.72–3.07)	0.472	
Yes	1.00	1.31 (0.88–1.96)	1.31 (0.86–1.99)	1.77 (1.18–2.66)	0.005	
Smoking						0.521
Never	1.00	1.11 (0.65–1.92)	1.19 (0.76–1.85)	1.77 (1.11–2.81)	0.010	
Former	1.00	1.75 (0.86–3.60)	1.50 (0.70–3.22)	1.59 (0.78–3.24)	0.496	
Current smoker	1.00	2.41 (0.61–9.61)	2.98 (0.98–9.11)	3.52 (1.15–10.73)	0.025	
Income to poverty ratio						0.799
< 1.30	1.00	1.12 (0.53–2.35)	1.40 (0.71–2.76)	1.75 (0.87–3.52)	0.055	
1.3–3.5	1.00	1.25 (0.70–2.23)	1.31 (0.79–2.16)	1.65 (0.96–2.84)	0.054	
> 3.5	1.00	2.01 (1.00–4.02)	1.64 (0.80–3.36)	1.78 (0.97–3.26)	0.259	
BMI level						0.887
Underweight/Normal	1.00	1.53 (0.73–3.23)	1.71 (0.85–3.45)	1.65 (0.79–3.41)	0.240	
Overweight	1.00	1.79 (0.84–3.84)	1.79 (1.00–3.22)	2.14 (1.05–4.35)	0.020	
Obese	1.00	1.16 (0.69–1.95)	1.06 (0.56–2.02)	1.63 (0.94–2.82)	0.105	

Model adjusted for age, gender and race, income-poverty ratio, leisure time physical activity, smoking, alcohol consumption, BMI, hypertension and diabetes

Stratification variables were not adjusted in the corresponding models

The *p* for interaction indicates that gender, hypertension and smoking do not significantly modify the relationship between blood lead and CVD mortality. Indeed, the *p*-values for interaction showed that the association between blood lead and CVD mortality did not significantly vary between men and women (*p* = 0.350), individuals with and without hypertension (*p* = 0.603), or different smoking status groups (*p* = 0.521).

### Population Attributable Fraction

The weighted prevalence of elevated blood lead (≥ 3.5 µg/L) is 10.3%. Comparing individuals with blood lead < 3.5 µg/L, those who had blood lead ≥ 3.5 µg/L had a HR of 1.48 (95%CI 1.30–1.68) for all-cause mortality and 1.29 (95%CI 1.02–1.63) for CVD mortality. The PAF of all-cause mortality and cardiovascular disease mortality associated with quartile 4 of blood lead were 13.0% and 14.2%, respectively. The corresponding figures were 13.2 and 11.7% associated with blood lead ≥ 3.5 µg/L Table 4.

**Table 4:** Supplemental Table Hazard ratio (95%CI) for mortality associated with elevated blood lead

	Blood lead ≥ 3.5 µg/L	
	No	Yes
All-cause mortality		
Model 1	1.00	1.74 (1.57–1.93)
Model 2	1.00	1.48 (1.30–1.68)
CVD mortality		
Model 1	1.00	1.50 (1.21–1.85)
Model 2	1.00	1.29 (1.02–1.63)

### Mediating Effect of Homocysteine Mediation on BLL-Related All-Cause and CVD Mortality

Odds ratios of CVD mortality according to total, direct, and indirect (homocysteine mediation) effects of BLL are reported in Table 5. Significant association between higher blood lead levels and increased risks of all-cause and CVD mortality. The indirect effect, mediated by homocysteine, significantly contributes to this associations. Indeed. In the highest quartile of BLL (OR 1.58 CI 1.48–1.68) with a 43.85 percent contribution to the total effect. Similarly, for CVD mortality, homocysteine contributes to about 42.33% of the total effect in Q4 (OR 1.37 CI 1.22–1.52).

**Table 5:** Odds ratios for different types of mortality according to total, direct, and indirect (homocysteine-mediated) effects on BLL

	Quartiles of blood lead						
	Q1	Q2		Q3		Q4	
	OR	OR	95%CI	OR	95%CI	OR	95%CI
*All-cause mortality*							
Total	Ref	1.47	1.28—1.68	1.80	1.55—2.09	2.83	2.40—3.34
Indirect	Ref	1.25	1.21—1.29	1.39	1.33—1.46	1.58	1.48—1.68
Direct	Ref	1.18	1.03—1.34	1.29	1.13—1.48	1.79	1.55—2.08
Percent contribution of indirect effect to total effect			57.95**		56.48**		43.85**
*CVD mortality*							
Total	Ref	1.40	1.01—1.94	1.65	1.14—2.41	2.09	1.47—2.96
Indirect	Ref	1.17	1.10—1.23	1.26	1.16—1.36	1.37	1.22—1.52
Direct	Ref	1.20	0.87—1.65	1.32	0.91—1.90	1.53	1.10—2.12
Percent contribution of indirect effect to total effect			45.6		45.47		42.33

Model 1 adjusted for age, gender and race, income-poverty ratio, leisure time physical activity, smoking, alcohol consumption, BMI, hypertension and diabetes

## Discussion

This prospective cohort study used NHANES data (1999–2006) to investigate homocysteine mediation effect on the association between blood lead levels and CVD mortality. Our findings demonstrated evidence that blood homocysteine levels mediate nearly half of the association between blood lead levels and CVD mortality. To our knowledge this is the first study demonstrating homocysteine mediation of the association between blood lead levels and CVD mortality.

During a mean 11.6 years of follow-up, BLL was positively associated with CVD and all-cause mortality. Indeed, our analysis revealed a dose-dependent increase in mortality rates, with the highest quartile BLL associated with an incidence rate of 23.9 per 1000 years. Furthermore, BLL was positively associated with increased homocysteine levels after adjusting for other covariates. Moreover, older, non-Hispanic White male participants had the highest BLL and homocysteine levels. Indeed, our data suggests that a non-Hispanic white male aged approximately 55 years of age with blood lead level of 2.1 µg/dL has a 32% higher risk of all-cause mortality, and, a 41% higher risk of CVD mortality than someone with a blood lead level of 0.7 µg/dL. Furthermore, our data demonstrates that low of blood lead levels were associated with an increased risk of cardiovascular mortality, and therefore, is increased risk to the general population. This is consistent with previous findings that low blood lead levels were associated with CVD mortality [12].

There is plenty of evidence that BLL is associated with increased homocysteine levels [28, 29]. Schafer et al. reported a positive association between BLL and homocysteine levels in a sample of 1,140 US adults [20]. In another prospective cohort study of 2280 American men, BLL was positively associated with homocysteine levels [29]. Several other studies investigating the association between BLL and homocysteine similarly detected a dose–response relationship between BLL and homocysteine [30]. Our data now further support the evidence base that BLL is likely to increase blood homocysteine, although of course these study designs do not prove causality.

The mechanism underlying the positive association between BLL and homocysteine concentrations has not yet been fully established. However, several possible mechanisms have been suggested. Lead may interact with sulfhydryl group-containing proteins [20]. It has also been shown that lead inhibits δ-aminolevulinic acid dehydratase (ALAD), an enzyme required for heme synthesis, thereby affecting the function of cystathionine β-synthase, an enzyme that catalyzes the first step of the transsulfuration pathway and the condensation of homocysteine to cystathionine, thereby leading to homocysteine accumulation. Another suggested mechanism is direct inhibition of homocysteine metabolism; since homocysteine contains a sulfhydryl group, lead may directly bind to this group, thereby inhibiting homocysteine and blood accumulation [30].

Our data show that after adjusting for age, gender, race, income-poverty ratio, leisure time physical activity, smoking, alcohol consumption, BMI, hypertension and diabetes., a BLL of 4.1 μg/dL was associated with CVD mortality. These data are consistent with Lanphear et al., who reported that BLL < 5 μg/dL was associated with an increased mortality risk [31]. Several other studies have reported a positive association between BLL and all-cause, CVD, and cancer-mortality at lower BLLs [32, 33]. Previous analyses of NHANES data demonstrated that BLL was associated with higher all-cause and CVD mortality [33]. Aoki et al. analyzed NHANES data from 1999 to 2010 and detected a linear association between BLL and increased CVD mortality [34]. Therefore, our data and previous reports of increased cardiac mortality rates in individuals with blood lead levels < 5 mg/dl suggest that no BLL can be considered safe.

Homocysteine has been reported as an independent risk factor for atherosclerosis development, leading to an increased risk of CVD including myocardial infarction, stroke, and peripheral vascular disease [35]. Elevated homocysteine levels may cause vascular damage via several mechanisms including endothelial dysfunction and damage to vascular smooth muscle [36]. Some proposed mechanisms for homocysteine-induced damage included increased oxidative stress, inhibition of nitric oxide synthesis, and proliferation of vascular smooth muscle cells [37]. Several studies have demonstrated a positive association between serum homocysteine levels and increased arterial stiffness [38, 39]. Ours is the first report that homocysteine indirectly mediates the association between BLL and cardiovascular mortality in a large representative sample of the US population, even after adjusting for multiple covariates.

## Limitations

There were some limitations associated with our study. Although it is established that blood lead levels increase homocysteine, in this study we didn’t seek to establish this. Therefore, bias due to unmeasured confounding factors may still be present. Another potential limitation is the presence of genetic abnormalities that are known to increase homocysteine levels, including CBS and MTHFR mutations.

## Conclusions

More than 40 percent of the increased CVD mortality related to blood lead was mediated by homocysteine levels in the US population.

## Data Availability

Data will be made available on request.
